# Sexual dysfunction among six months postpartum women in north-eastern Malaysia

**DOI:** 10.1371/journal.pone.0284014

**Published:** 2023-04-05

**Authors:** Ying Ying Ng, Rosediani Muhamad, Imran Ahmad

**Affiliations:** Department of Family Medicine, School of Medical Sciences, Universiti Sains Malaysia, Kubang Kerian, Kelantan, Malaysia; University of the Witwatersrand, SOUTH AFRICA

## Abstract

Female sexual dysfunction (FSD) is a common problem among postpartum women. However, little is known about this topic in Malaysia. This study aimed to determine the prevalence of sexual dysfunction and its associated factors in postpartum women in Kelantan, Malaysia. In this cross-sectional study, we recruited 452 sexually active women at six months postpartum from four primary care clinics in Kota Bharu, Kelantan, Malaysia. The participants were asked to fill in questionnaires consisting of sociodemographic information and the Malay Version of the Female Sexual Function Index-6. The data were analyzed using bivariate and multivariate logistic regression analyses. With a 95% response rate, the prevalence of sexual dysfunction among sexually active, six months postpartum women was 52.4% (n = 225). FSD was significantly associated with the older husband’s age (p = 0.034) and lower frequency of sexual intercourse (p<0.001). Therefore, the prevalence of postpartum sexual dysfunction in women is relatively high in Kota Bharu, Kelantan, Malaysia. Efforts should be made to raise awareness among healthcare providers about screening for FSD in postpartum women and for their counseling and early treatment.

## Introduction

The American Psychiatric Association defines female sexual dysfunction (FSD) as a disturbance in the process that characterizes the sexual response cycle or pain associated with sexual intercourse [1]; these prevent individuals from experiencing satisfaction with sexual activity [2]. Sexual dysfunction is a complex clinical entity that involves biopsychosocial components and cultural and religious factors that play a role in its development [3]. Among Malaysian women, sexual problems tend to be ignored, and openly discussing this issue is discouraged owing to culture and religion [3]. Furthermore, the conservative sociocultural standards in Malaysian society also make women feel embarrassed when discussing sexual issues, as these topics are considered taboo [3]. This can negatively affect the well-being of women and lead to interpersonal difficulties, marital dissatisfaction, divorce, and reduced quality of life [4].

The Diagnostic and Statistical Manual of Mental Disorders, Fifth Edition, simplified the classification of FSD into three groups (instead of five) in the last version, namely, female sexual interest/arousal disorder, female orgasmic disorder, and genital pelvic pain/penetration disorder [5]. Of every 10 women in the reproductive age group, four have at least one domain of FSD [6]. In Malaysia, the prevalence of sexual dysfunction ranges from 25.8%–29.6% among women receiving primary care; low sexual desire and arousal were the most affected domains. Several factors have been reported to be significantly associated with this, such as older age, longer marriage duration, having more children, and low frequency of sexual intercourse [3, 7]. Additionally, more prevalence studies have been conducted among Malaysian women with medical problems, including rheumatoid arthritis (29.4%), diabetes mellitus (26.4%), psoriasis (20.3%), and hypertension (18.2%) [8–11]. However, at the time of this study, data on sexual dysfunction in postpartum women were limited.

During the postpartum period, women’s sexual function might be influenced by trauma to reproductive organs, vaginal bleeding, constraints caused by a newborn, lethargy, changes in body shape, and physical discomfort, such as breast tenderness [12]. The childbirth process affects women’s bodies, especially their genitalia, and these changes make women hesitant to resume sexual intercourse [12]. More than 50% of women experience pain during their first intercourse after birth, and 41% still experience dyspareunia three months postpartum [13]. Although most women resume sexual intercourse by 6–8 weeks after childbirth, it might take a year to return to pre-pregnancy levels of sexual activity [14]. The time to resume sexual intercourse during the postpartum period varies according to culture. In Malaysia, as the majority of the population is Malay Muslim, women primarily practice longer confinement periods, which are usually 40–44 days. Furthermore, sexual activity is prohibited by religion during the period of “nifas” (a period following delivery when the mother continues to pass lochia) [15]. During the confinement period, Malaysian women participate in typical confinement practices, including the consumption of traditional herbs (usually prepared from plant-derived spices, local roots, honey, ginger, dried fruits, and garlic), “bertungku” (also known as hot compression, a kind of point massage that uses heated materials to reduce muscle spasm), “barut” (an abdominal binder similar to a corset), body massage and external herbal application [15]. Most Malaysian traditional postpartum practices aim to preserve the balance among the four body elements: earth, fire, water, and wind. Additionally, they intend to restore normal sexual function and reproductive organs, promote wound healing, restore mothers’ energy, and return mothers’ pre-pregnancy weight. It is believed that delivery is considered a ‘cold’ state; therefore, proper care is essential throughout the confinement to balance the ‘hot’ and ‘cold’ states within the body through heat therapy, alteration of diet, and lifestyle changes [15].

Childbirth is a vulnerable period for women in which they encounter various new changes, concerns, and problems. Women who experience sexual problems after childbirth may be at greater risk of experiencing marital dissatisfaction, depressive symptoms, or other medical problems [13, 16]. Postpartum sexual function is a crucial issue for couples especially in the first sexual intercourse, to establish an intimate relationship [17]. Furthermore, sexual problems have been reported to be the second leading cause of divorce among Muslim couples [3]. Healthcare professionals do not always have the awareness, knowledge, and skills to deal with postnatal sexual problems. Postpartum medical advice about sexuality usually focuses only on contraception [13]. Postpartum women need sensitive professional counseling and reassurance about their body, and their sexual life after childbirth. Thus, this study aimed to determine the prevalence and factors associated with sexual dysfunction among postpartum women.

## Methods

### Participants and design

This cross-sectional study was conducted among 429 women at six months postpartum in four primary care clinics with family medicine specialists in Kota Bharu, Kelantan, Malaysia, from February to November 2019. Women who came for their 6-month-old child’s immunization at the primary care center were identified. Women at six months postpartum were included in this study to reflect the long-term consequences of morbidity [18]. The other inclusion criteria were: women aged ≥18 years, singleton childbirth at term, and living with a husband. Women with psychosis, in same-sex relationships, who had not yet resumed sexual intercourse after delivery, pregnant women, and non-Malaysian women were excluded from this study.

### Sample size

The sample size to determine the prevalence of sexual dysfunction among postpartum women was calculated using a single-proportion formula as follows:

$$\frac{n={\left(Z\right)}^{2}\times P(1-P)}{\Delta^{2}}$$

where n = number of samples; Z = standard normal deviate, which is 1.96 (95% confidence level); P = prevalence rate 43.5% (0.435) from a previous study by De Lima Holanda et al. [19]; = precision rate of 5% (0.05).

The minimum sample size was 378; and after considering a 20% nonresponse rate, the calculated sample size was 452.

### Instruments

The questionnaire used in this study consisted of two parts. The first part included the participant’s information, which consisted of sociodemographic data, general clinical well-being, and marital and sexual profiles. The second part was the Malay version of the Female Sexual Function Index-6 (FSFI-6).

#### Sociodemographic data, clinical and general well-being, marital and sexual profile

This section consists of the patient’s sociodemographic data (age, family income, education level, and occupation), clinical and general well-being (number of children, mode of delivery, parity, breastfeeding, presence of chronic medical illness, such as diabetes mellitus, hypertension, chronic kidney disease, heart disease, and cancer, confinement practices, such as consuming herbs, having a body massage or heat therapy “bertungku” during the first 40 days postpartum), and marital and sexual profile (husband’s age, time of resumption of sexual intercourse after childbirth, duration of marriage, and frequency of sexual intercourse).

#### Malay Version Female Sexual Function Index-6 (FSFI-6)

The FSFI-6 is a questionnaire used to assess women’s sexual function during the previous four weeks. It is a simplified version of the FSFI-19 [20] and has been validated among 200 women attending outpatient clinics for sexual and reproductive health [21]. The FSF1-6 includes one question for each of the six domains: desire, arousal, lubrication, orgasm, satisfaction, and pain. Desire and satisfaction items were rated from 1–5, and the other domains’ items were rated from 0–5. The total score ranges from 2–30, with lower scores indicating poorer sexual functioning. The receiver operating characteristic (ROC) curves of the FSFI-6 showed that women who scored ≤19 points were classified as screened positive for FSD. Using 19 as the cut-off, the sensitivity and specificity of the test were 0.93 and 0.94, respectively; Cronbach’s alpha was 0.789 [21]. The Malay version of the FSFI-6 was tested and validated in 128 breast cancer patients enrolled in another study, with a Cronbach’s alpha of 0.93 [22]. The FSFI-6 is a faster screening tool for FSD and is easy to use during studies or outpatient visits.

### Procedure

Based on attendance at the Maternal Child Health Clinic in the four primary health clinics with family medicine specialists, a nonproportionate systematic random sampling with a 1:2 ratio was applied. Participants were identified based on the eligibility criteria for the study; then, they were briefed and invited to participate. Written consent was obtained once participants voluntarily agreed. Self-administered questionnaires were distributed, and it took approximately 10–15 minutes to complete the questionnaires in a private space. During this time, the researcher was available for any clarification of the questionnaire, which was collected once completed, and participants were reassured of their information confidentiality. Those who scored positive for FSD on the questionnaire were immediately notified of the results. Those who desired further FSD management were referred to a psychiatrist, while those who did not were given the psychiatric clinic’s contact information in case, they wished to seek treatment in the future.

This study was approved by the Research and Ethics Committee of the Universiti Sains Malaysia (USM/JEPeM/18080359) and the Medical Research Ethics Committee of the Ministry of Health of Malaysia (NMRR-18-2551-43304).

### Data analysis

Data were analyzed using IBM SPSS Statistics for Windows, version 26.0 (IBM Corp., Armonk, NY, USA). Descriptive analyses were used to describe all categorical (frequency and percentage) and numerical (mean and standard deviation [SD]) variables. Bivariate and multivariate logistic regression analyses were performed to screen for potential factors associated with FSD. All variables with p-value ≤0.25 for bivariate logistic analysis were included in the multivariate logistic regression analysis to determine the factors associated with FSD [23], whereas other confounders in the model were controlled. The dependent variable was FSD (categorized as FSD with a score of FSFI-6 ≤19 and non-FSD with a score of FSFI-6 >19), whereas the independent variables were sociodemographic characteristics. The results were presented as an odds ratio (OR) with a 95% confidence interval (CI). A p-value <0.05 following multivariate logistic analysis was considered statistically significant.

## Results

A total of 452 women who attended the clinic were invited to participate in this study. All participants had resumed sexual activities six months post-delivery. However, 23 women were excluded due to refusal to participate or incomplete questionnaires. The response rate was 95%, and 429 respondents were included in further analyses.

The sociodemographic, clinical, and marital data of the participants are summarized in Table 1. The mean age of participants was 30.9 years (SD = 5.55), and the mean household income was US$593 (SD = 428.5); half of them had attained secondary school education (53.4%) and were housewives (54.8%). Approximately two-thirds of the participants were multiparous (68.1%) and had delivered vaginally with sutures (71.3%). The rates of mixed feeding and exclusive breastfeeding were comparable at 45.2% and 43.4%, respectively. Most participants had no chronic medical illness (97%), and they underwent confinement practices, such as consuming herbs, having a body massage or heat therapy “bertungku”, within 40 days after delivery (79%). The mean age of their husbands was 33.9 years (SD = 6.5), the mean duration of marriage was 6.7 years (SD = 5), and the mean number of weeks of resumption of first sexual intercourse after delivery was 12.9 weeks (SD = 3.4). Also, approximately 54.8% of the participants had sexual intercourse once a week or more.

**Table 1:** Sociodemographic and clinical data of the participants (n = 429).

Variables	Mean (SD)	n (%)
**Sociodemographic data**		
Age (years)^a^	30.9(5.55)	
Household income (US$/month)^a^	593.0(428.5)	
Education level		
Nil, primary or secondary		240(55.9)
Tertiary		189(44.1)
Occupation		
Housewife		235(54.8)
Self-employed		54(12.6)
Government/private sector		140(32.6)
**Clinical and general wellbeing**		
Number of children ^a^	2.4(1.39)	
Mode of delivery		
Vaginal delivery without suture		49(11.4)
Vaginal delivery with suture		306(71.3)
Caesarean section		74(17.2)
Parity		
Primiparous		137(31.9)
Multiparous		292(68.1)
Breastfeeding		
No breastfeeding		49(11.4)
Partially breastfeeding		194(45.2)
Exclusive breastfeeding		186(43.4)
Chronic medical illness		
Present		13(3.0)
Absent		416(97.0)
Confinement practises		
Present		339(79.0)
Absent		90(21.0)
**Marital and sexual profile**		
Husband’s age (years)^a^	33.9(6.5)	
Resumption of sexual intercourse (weeks)^a^	12.9(3.4)	
Duration of marriage (years)^a^	6.7(5.0)	
Frequency of sexual intercourse (weeks)		
Every once a week or more		235(54.8)
Once fortnightly		127(29.6)
Once a month		67(15.6)

FSD, female sexual dysfunction

^a^ mean (standard deviation)

Of the 429 respondents, 225 screened positive for FSD, a total prevalence of 52.4%. The mean FSD and its subdomains’ scores are presented in Table 2.

**Table 2:** Female sexual dysfunction and subdomain scores.

Variables	Mean (SD)	95% CI
FSFI-6 score	19.4 (3.62)	19.07, 19.75
Subdomains		
1. Desire	2.9 (0.58)	2.90, 3.01
2. Arousal	3.0 (0.59)	2.97, 3.08
3. Lubrication	3.0 (1.20)	2.87, 3.10
4. Orgasm	3.4 (1.21)	3.32, 3.55
5. Satisfaction	3.7 (1.00)	3.64, 3.83
6. Sexual pain	3.3 (1.30)	3.15, 3.40

CI, confidence interval; FSFI-6, Female Sexual Function Index-6; SD, standard deviation

The bivariate logistic regression analysis of the factors associated with FSD is shown in Table 3. Education level, mode of delivery, husbands’ age, duration of the marriage, and frequency of sexual intercourse were statistically significant after bivariate analysis. Then, a multivariate logistic regression analysis was performed using the presence of FSD as the dependent variable and all parameters that were significantly associated with FSD in the bivariate analysis. The multivariate analysis models were the same regardless of the variable selection method applied (forward or backward logistic regression). Only the husband’s age and frequency of sexual intercourse remained statistically significant (p<0.05) in the multivariate logistic regression analysis (Table 4). Husband age older than 1 year had 1.03 odds of sexual dysfunction of the women (adjusted OR [aOR] 1.03; 95% CI 1.00–1.07; p = 0.034). However, it had a small clinical effect. The frequency of sexual intercourse once every two weeks (aOR 2.09; 95% CI 1.34–3.25; p = 0.001) and once a month (aOR 3.89; 95% CI 2.13–7.13; p<0.001) was approximately two and three times the odds of sexual dysfunction, respectively, in comparison to those who had sexual intercourse once or more per week.

**Table 3:** Associated factors for female sexual dysfunction using bivariate logistic regression.

Variable	Crude *OR* ^a^	(95% CI^b^)	Wald stat^c^	*P* value
**Socio-demographic**				
Age(years)	1.01	(0.96,1.04)	0.24(1)	0.63
Household income (US$/month) Education level	1.00	(1.00,1.00)	1.06(1)	0.30
Nil or primary, secondary	1			
Tertiary	0.71	(0.48,1.04)	3.15(1)	0.08
Occupation				
Housewife	1			
Self-employed	0.91	(0.50,1.64)	0.10(1)	0.76
Government/private sector	1.05	(0.69,1.60)	0.05(1)	0.82
**Clinical and general wellbeing**				
Number of children	1.03	(0.89,1.18)	0.14(1)	0.71
Mode of delivery				
Vaginal delivery without suture	1			
Vaginal delivery with suture	1.31	(0.72,2.20)	0.77(1)	0.38
Caesarean section	1.90	(0.92,3.96)	2.98(1)	0.08
Parity				
Primiparous	1			
Multiparous	1.13	(0.75,1.70)	0.35(1)	0.55
Breastfeeding				
No breastfeeding	1			
Partially breastfeeding	1.16	(0.62,2.16)	0.20(1)	0.66
Exclusive breastfeeding	1.19	(0.63,2.23)	0.28(1)	0.60
Chronic medical illness				
Absent	1			
Present	1.47	(0.47,4.56)	0.44(1)	0.51
Confinement practises				
Absent	1			
Present	0.81	(0.51,1.29)	0.81(1)	0.37
**Marital and sexual profile**				
Husband’s age (years)	1.03	(1.00*,1.06)	2.74(1)	0.10
Resumption of sexual intercourse (weeks)	1.02	(0.97,1.08)	0.52(1)	0.47
Duration of marriage (years)	1.03	(0.99,1.07)	1.70(1)	0.19
Frequency of sexual intercourse				
Once a week or more	1			
Once fortnightly	2.01	(1.30,3.12)	9.74(1)	<0.001
Once a month	3.68	(2.02,6.69)	18.1(1)	<0.001

FSD, female sexual dysfunction.

^a^ Crude odds ratio

^b^ Confidence interval

^c^ Wald statistic

**Table 4:** Associated factors for female sexual dysfunction using multivariate logistic regression.

Variables	Adj. *OR* ^a^	(95% *CI* ^b^)	Wald stat ^c^	*P* value
Husband’s age(years)	1.03	(1.00*,1.07)	4.48(1)	0.034
Frequency of sexual intercourse				
Once a week or more	1			
Once fortnightly	2.09	(1.34,3.25)	10.59(1)	0.001
Once a monthly	3.89	(2.13,7.13)	19.39(1)	<0.001

^a^ Adjusted odds ratio

*1.002

^b^ Confidence interval

^c^ Wald statistic

*Note*. No significant interaction; no multicollinearity problem; model assumptions met.

Model fitness was tested using the Hosmer–Lemeshow goodness-of-fit test, classification table, and area under the ROC curve. The p-value for the Hosmer–Lemeshow test was not significant (p = 0.794), the classification table showed that FSD was correctly predicted in 62.2% of cases, and the area under the ROC curve was 0.652. The preliminary model was accepted for the final model based on the Hosmer–Lemeshow goodness-of-fit test and ROC curve, as the classification table did not produce good values.

## Discussion

Sexual dysfunction has a crucial effect on the quality of life and intermarital relationships between couples. During the postpartum period, women experience numerous physical and psychological changes that may affect their sexual function. According to Acele et al., the prevalence of sexual dysfunction was higher during the postpartum period than before pregnancy [12]. Postpartum sexual function is important for couples, especially for the first instance of postpartum sex, to establish intimate relationships [17]. Our study was conducted to evaluate the female sexual function at six months postpartum. The cultural practice of abstaining from sexual intercourse during the confinement period in Malaysia varies [3]; however, one study showed that most women resumed sexual intercourse within six months postpartum [24].

### Prevalence of sexual dysfunction among women after childbirth

Our study showed that the prevalence of FSD in sexually active married women at six months postpartum was 52.4%. This result is comparable with another local study conducted among women at four to six months postpartum in urban primary care settings in Malaysia, where the prevalence was 57% [25]. However, some developed countries had reported an even higher prevalence of FSD. For example, a study in Australia reported that sexual dysfunction was observed in 64% of women during the first year after childbirth [26]. Furthermore, in a sample of Iranian women at eight weeks to eight months postpartum, it was even higher at 76.3% [27]. Compared with other ASEAN countries, sexual dysfunction among six months postpartum women was demonstrated in approximately 31% and 34.2% in Thailand [28] and China [29], respectively. Different sociodemographic backgrounds, cultural backgrounds, and beliefs may contribute to the different prevalence of sexual dysfunction among postpartum women [30]. This variation may also be due to differences in the time postpartum women were studied, such as four to six months or a year after childbirth. Sexual dysfunction is highly prevalent in postpartum women worldwide.

In the postpartum period, many changes can affect a woman’s sexual response cycle, including pain during intercourse, lack of sexual desire, vaginal dryness, and failure to reach orgasm [31]. This study showed that the mean score for desire problem was 2.9, which was the lowest among the six domains, followed by a mean score of 3 for lubrication and arousal problems. One factor is the high prolactin hormone level in breastfeeding mothers, which can lead to decreased estrogen hormone levels and contribute to low sexual desire and decreased vaginal lubrication [32]. This is in line with the results of the present study, as the majority of participants (88.6%) breastfed their babies. Breastfeeding can also cause tiredness, changes in a mother’s sexual desire, and changes in a couple’s relationship, as nursing is a time-consuming activity that may interfere with intimate emotional exchanges between partners [26]. Parents who are fatigued and frequently suffer from sleep deprivation, such as denoting the time and effort to care for a newborn and loss of sexual desire, were factors that interfered with couples’ sexual lives and relationships [33].

### FSD-associated factors

In this study, half of the women (54.3%) were sexually active at least once per week. Regression analyses showed that women who had sexual intercourse less frequently than once a week had a higher risk of sexual dysfunction. According to this study model, for women who had sexual intercourse every two weeks, the OR for FSD was 2.09 times higher than that for women who had sexual intercourse once a week or more. These findings are consistent with those of a study in Australia indicating that a low frequency of sexual activity is associated with problems in sexual function [26]. The weekly frequency of sexual intercourse decreased in 75% of couples, which was caused by the time dedicated to the child and dyspareunia [34]. Furthermore, in Malaysia, it is common to have small children sleep with their parents in the same bedroom up to the child’s schooling age [35]. In contrast, women with sexual dysfunction are more likely to have less frequent or no sexual activity than women without it [26]. Another explanation is that a higher frequency of sexual activity can improve intimate relationships between couples, leading to lower a risk of sexual dysfunction. This has also been found in another study that reported a correlation between the degree of sexual satisfaction and changes in the frequency of intercourse [36].

Additionally, this study found that the husband’s age was significantly associated with FSD. This is probably due to multiple factors related to increasing age, such as a decline in health and increased use of medicines, which may affect sexual function [7]. Another local study reported a similar finding: women married to older husbands had an increased probability of developing FSD [3]. This finding suggests that sexual dysfunction in women can be affected by husbands’ performance. Male partners’ sexual dysfunction is a risk factor for FSD. Female partners reported significantly lower sex drive and reduced in sexual activity [37]. However, the present study did not investigate the details of husbands’ risk factors for sexual dysfunction, such as medical conditions and smoking. Further studies could examine the factors of sexual dysfunction in husbands and their effects on women’s sexual function.

The results of the present study showed that the mode of delivery was not significantly associated with FSD, although many studies have shown this association, especially between FSD and vaginal delivery with sutures or episiotomy [19, 38, 39]. The mean number of weeks between delivery and first sexual intercourse encounter was 12.9 weeks, which was comparable with some cultural beliefs of sexual abstinence for 100 days to allow proper healing after childbirth [15]. Some sexual problems that occur after vaginal delivery can be due to early initiation of sexual intercourse and incomplete healing of the episiotomy wound [40]. A meta-analysis conducted in 2017 showed that the mode of delivery—cesarean or vaginal delivery—did not affect sexual function [41]. Therefore, there are mixed findings in the literature on the relationship between the mode of delivery and sexual function. Owing to concerns regarding the rise in maternal requests for cesarean section in recent years, these observations should be further investigated in larger prospective longitudinal studies. Furthermore, future studies should be conducted to survey women’s perceptions and concerns about the mode of delivery that affects sexual function after childbirth.

Additionally, our study found no significant association between FSD and age, education level, employment, parity, or breastfeeding, which is supported by another study from Japan [42]. According to some researchers, breastfeeding mothers experience increased sexual arousal and libido. However, some studies have reported lower sexual activity in women who breastfeed, which can be attributed to a decline in estrogen levels, leading to reduced vaginal lubrication and possible dyspareunia [43, 44]. Further studies with larger sample sizes are required to investigate this association.

This study has some limitations. First, these results cannot be generalized to the Malaysian population because most participants in Kelantan are Malays; therefore, this does not reflect the true ethnic distribution of Malaysia. The results are also not generalizable to all postpartum women, as this study did not include women who did not bring their 6-month-old children for immunization, those who were non-married, and those with psychosis. Second, a causal relationship could not be established because a cross-sectional study was conducted. Finally, we did not include the confounding effects of other variables, such as intimate partner violence and the quality of the couple’s relationship.

To our knowledge, this is the first study to use a simplified version of the FSFI-6 questionnaire to screen for FSD among postpartum women. It is important to remember that this measure is intended to assess sexual function and identify women with poorer function rather than to establish a clear diagnosis of FSD. Additionally, as the study was a self-administered questionnaire, the participants were expected to provide more reliable answers, as discussing sexuality openly is perceived as taboo. However, there is still a risk of information bias, even with the use of a self-reporting methods.

Future research studies should include a larger sample and prolonged postpartum follow-up to verify these findings and assess the effect of other demographic, cultural, psychological, and couple variables that may influence women’s sexual functioning in the postpartum period.

## Conclusions

Our study highlighted that a significant number of women at six months postpartum screened positive for sexual dysfunction, underscoring the importance of the current understanding of the effects of FSD on our population. Postpartum sexual health is a common concern but seldom discussed during prenatal or postpartum care [45]. Most women who have sexual issues do not seek professional help due to embarrassment and preoccupations with their newborn(s) [46]. Other potential barriers to conversations regarding sexual health issues in primary care include lack of knowledge, cultural taboos, and restriction of time allotted for each medical appointment [47]. Although sexual issues among women have begun to receive attention in Western nations, this is not the case in Malaysia. Traditionally, Malay women are not supposed to comment on their sexual desire or dissatisfaction because Islam forbids sexual disclosure unless for medical purposes [3]. Women have the right to receive treatment and reassurance, as this has been shown to affect the relationship between the couple and the mother’s psychological status. Therefore, healthcare providers play a significant role in assisting postpartum women by screening for FSD with a simple questionnaire (e.g., FSFI-6) and initiating a conversation about their sexual health as a protocol-based postnatal follow-up. Prenatal visits are also an ideal time for healthcare providers to address these concerns with expectant mothers and offer helpful advice that may be referenced at the follow-up postpartum appointment. Efforts should be made to engage women and their partners, and appropriate referrals for multidisciplinary support should be made as needed.

## Supporting information

S1 Data (XLSX)
